# Overview of human health in the Arctic: conclusions and recommendations

**DOI:** 10.3402/ijch.v75.33807

**Published:** 2016-12-13

**Authors:** Shawn Donaldson, Bryan Adlard, Jon Øyvind Odland

**Affiliations:** 1 Population Biomonitoring Section, Health Canada, Ottawa, ON, Canada; 2 Department of Community Medicine, Faculty of Health Sciences, University of Tromsø – The Arctic University of Norway, Tromsø, Norway

**Keywords:** AMAP, Arctic, human health, biomonitoring, contaminants, risk, environmental change

## Abstract

This article is intended to provide an overview of the key conclusions, knowledge gaps and key recommendations based on the recent 2015 Arctic human health assessment under the Arctic Monitoring and Assessment Program. This assessment was based primarily on data from human health monitoring and research studies and peer-reviewed literature published since the last assessment in 2009.

Over the last 25 years, Arctic Monitoring and Assessment Program (AMAP) has documented levels of contaminants, and the health impacts of contaminants and other stressors on human populations living in the Arctic. The AMAP recently completed a human health assessment (1), which built on the conclusions of previous assessments (2–4) and provided a synthesis of Arctic monitoring data, human health research, risk description and risk communication issues. This assessment also included a novel chapter focused on adaptation to the environmental impacts of climate change. Here, we review the key conclusions, knowledge gaps and recommendations for each of the key topic areas.

## Biomonitoring

Biomonitoring is important for understanding past and current human exposure to contaminants. Long-term monitoring in some Arctic regions has allowed for time trend comparisons to be made, which have found that most persistent organic pollutants (POPs) and metals have declined across many parts of the Arctic. For example, levels of POPs have declined by an average of 80% and mercury by 59% over the past 20 years of monitoring in Nunavik. Despite this downward trend, some contaminants such as mercury remain high among certain populations including some Inuit in Canada and Greenland, and still exceed blood guidance levels in some of these regions. Baseline levels of some POPs recently added to the Stockholm Convention, such as certain brominated flame retardants and perfluorinated contaminants, have been measured in several Arctic regions; however, these contaminants do not behave like most other POPs, such as organochlorines, and more data are required to evaluate the routes of exposure and describe spatial and temporal trends in human populations. Further monitoring of contaminants is still needed in all Arctic regions to determine whether declining trends of some POPs continue, in addition to monitoring of new Stockholm POPs for which there remains limited data available. Biomonitoring data should also be generated in a coordinated, international approach, as this will provide globally comparable data sets to aid in understanding trends.

## Human health effects

The results from several cohort and research studies have indicated human health effects related to current and past exposure to POPs and/or metals such as mercury and lead. Cohort studies in Nunavik and the Faroe Islands have documented neurobehavioral effects in children related to exposure to methylmercury and lead, immunological effects from organochlorines (high incidence of infectious diseases in Nunavik children) and perfluorinated compounds (reduced vaccine response in Faroese children), and cardiovascular effects related to mercury although results are not consistent in all regions. Studies have suggested that exposure to POPs is associated with increased risk of developing type 2 diabetes, such as one recent study that investigated the influence of POPs on type 2 diabetes in a group of Faroese septuagenarians. Despite this, current knowledge remains limited. Additional studies are needed to better understand recently observed health effects and risks associated with current levels of exposure in the Arctic. Traditional foods can provide beneficial nutrients and future studies on the effects of mercury should include and incorporate an analysis of seafood nutrients to avoid underestimating the associations between childhood deficits and methylmercury exposure. Studies should focus more on reporting descriptive statistics about the distributions of response variables and explanatory variables, which are needed when summarizing and meta-analysing the magnitude of effects of contaminants exposure on health outcomes. In addition, study protocols should be harmonized wherever practical to improve opportunities for comparing contaminant levels and effects data between different regions of the world.

## Risk description

Risk assessment is an important tool in the overall process of protecting the health of Arctic residents. Assessing exposure and hazard identification are critical components, and a number of different methods are available. Biomonitoring data provide valuable data on the sum of exposure from various exposure routes; however, translating concentrations of contaminants in blood into specific health effects remain a challenge.

## Risk communication

In the Arctic, the most significant route of exposure for POPs is from the consumption of traditional foods, however dietary advice can be complex for several reasons. Communicating the risks and benefits associated with dietary choices of both traditional and imported foods must be done carefully and in partnership with affected communities, taking into account a wide range of factors (social, economic and cultural) to ensure that advice is culturally appropriate. Vulnerable individuals (such as elders, women of child-bearing age, infants and children) in communities should be identified as this can improve the development of population-specific adaptation strategies to reduce their risks of exposure to dietary contaminants and disease. Consumption of most traditional foods is still recommended as a healthy food choice, although preference is given to foods that are lower on the food chain, containing lower concentrations of contaminants.

## Adaptation in a changing Arctic environment

The Arctic environment is changing and potential risks to human health include environmental contaminants, climate change and zoonotic diseases. More research and biomonitoring that is linked to environmental changes is required to better understand what impact climate-mediated environmental changes are having on Arctic populations, including the availability and accessibility of wildlife as traditional foods. Region-specific adaptation strategies need to be developed at the community level, which also identify vulnerable individuals, and address contaminants, climate change and emerging zoonotic diseases, as well as interactions between these factors.

## Future activities

The AMAP Human Health Assessment Group will be developing a strategic blueprint to outline future research priorities and activities, based on the findings of this AMAP assessment. This future work will build on previous AMAP accomplishments and seek to address the key knowledge gaps identified in the Arctic.
